# Graphics for relatedness research

**DOI:** 10.1111/1755-0998.12674

**Published:** 2017-05-12

**Authors:** Iván Galván‐Femenía, Jan Graffelman, Carles Barceló‐i‐Vidal

**Affiliations:** ^1^ Department of Computer Science, Applied Mathematics and Statistics Universitat de Girona Girona Spain; ^2^ Disease Genomics‐GCAT Group Germans Trias Health Research Institute (IGTP)‐Program of Predictive and Personalized Medicine of Cancer (PMPPC), Can Ruti Campus Badalona Barcelona Spain; ^3^ Department of Statistics and Operations Research Universitat Politècnica de Catalunya Barcelona Spain; ^4^ Department of Biostatistics University of Washington Seattle WA USA

**Keywords:** compositional data analysis, identical by state/descent, isometric log‐ratio, microsatellite, relatedness, ternary diagram

## Abstract

Studies of relatedness have been crucial in molecular ecology over the last decades. Good evidence of this is the fact that studies of population structure, evolution of social behaviours, genetic diversity and quantitative genetics all involve relatedness research. The main aim of this article was to review the most common graphical methods used in allele sharing studies for detecting and identifying family relationships. Both IBS‐ and IBD‐based allele sharing studies are considered. Furthermore, we propose two additional graphical methods from the field of compositional data analysis: the ternary diagram and scatterplots of isometric log‐ratios of IBS and IBD probabilities. We illustrate all graphical tools with genetic data from the HGDP‐CEPH diversity panel, using mainly 377 microsatellites genotyped for 25 individuals from the Maya population of this panel. We enhance all graphics with convex hulls obtained by simulation and use these to confirm the documented relationships. The proposed compositional graphics are shown to be useful in relatedness research, as they also single out the most prominent related pairs. The ternary diagram is advocated for its ability to display all three allele sharing probabilities simultaneously. The log‐ratio plots are advocated as an attempt to overcome the problems with the Euclidean distance interpretation in the classical graphics.

## 1 Introduction

Statistical methods for the analysis of the genetic relationships between individuals of a population are of great relevance for molecular ecology (Blouin, 2003). Studies of relatedness are crucial for studying population structure, evolution of social behaviour, genetic diversity, quantitative genetics, etc. It is known that the estimation of quantitative genetic parameters in wild populations is less biased and more precise if we dispose of pedigree information (Bérénos, Ellis, Pilkington, & Pemberton, 2014). The role of relatedness for selective breeding is also recognized. Loughnan, Smith‐Keune, Jerry, Beheregaray, and Robinson (2016) recommend low levels of relatedness and high levels of neutral genetic diversity to form a base population for selective breeding. The exclusion of duplicated individuals and close relatives is a previous quality control filter used in studies of population structure (Gonder et al., 2015). Relatedness estimation is also important for conservation programmes, and the performance of several estimators has been compared in that context (Oliehoek, Windig, van Arendonk, & Bijma, 2006). It plays an important role in structuring societies with fusion–fission dynamics (Croft et al., 2012; Snyder‐Mackler, Alberts, & Bergman, 2014; Spencer et al., 2015), can bias estimates of allele frequencies (Hansen, Nielsen, & Mensberg, 1997) and violates the assumption of independent individuals in trait‐gene association studies (Foulkes, 2009). Thus, statistical methods that can verify documented or uncover undocumented family relationships in the database are important tools in molecular ecology.

Relatedness investigations can be carried out in an entirely numerical manner by inspecting estimated IBS (identity by state) and IBD (identity by descent) probabilities, likelihood ratios or confusion matrices (Boehnke & Cox, 1997; Epstein, Duren, & Boehnke, 2000). Graphics greatly facilitate the interpretation of the results of relatedness studies and are increasingly being used (Abecasis, Chemy, Cookson, & Cardon, 2001; Pemberton, Wang, Li, & Rosenberg, 2010; Rosenberg, 2006). The main aim of this article was to summarize the state of the art of the graphical methods used in relatedness research. Relatedness investigations are based on allele sharing, and we will consider techniques that use IBS alleles as well as those using IBD alleles. A plot of the means against the standard deviations of the IBS counts is a powerful tool to detect relatedness (Abecasis et al., 2001). We explore this tool in detail and establish the domain of this graphic from a mathematical point of view. Plots of the proportions of markers with 0, 1 or 2 IBS counts (*p*_0_, *p*_1_ or *p*_2_) are often used to assess the existence of family relationships (Rosenberg, 2006). Nevertheless, if the researcher is interested in identifying the degree of relatedness, plotting the probabilities of sharing 0, 1 or 2 IBD alleles (*k*_0_, *k*_1_ or *k*_2_) is the best strategy. The IBD probabilities depend directly on relatedness and enable us to accurately infer the type of relationship. In addition to the former graphical methods, we propose to use graphics from compositional data analysis (CoDA) for both IBS and IBD allele sharing studies. Due to the fact that the proportions (*p*_0_, *p*_1_, *p*_2_) and the probabilities (*k*_0_, *k*_1_, *k*_2_) are constrained to sum to one, it is possible to apply all the graphical and analytical CoDA techniques introduced by Aitchison (1986) and developed posteriorly by Pawlowsky‐Glahn and Buccianti (2011). Two graphics, commonly used in CoDA, are of particular relevance for relatedness studies: the ternary diagram (also known as a *de Finetti* diagram in genetics) and a scatterplot of log‐ratios. We show the ternary diagram to be useful for plotting the proportions of the IBS counts and for plotting the estimated Cotterman coefficients (IBD probabilities). Moreover, the theoretical IBD sharing probabilities for the standard family relationships can be used as reference points in the ternary diagram (Thompson, 2000). Furthermore, the CoDA techniques allow us to introduce the isometric log‐ratio coordinates (ilr‐coordinates) of the vectors ***p*** = (*p*_0_, *p*_1_, *p*_2_) and ***k*** = (*k*_0_, *k*_1_, *k*_2_), which we can represent in a scatterplot. These ilr‐coordinates allow us to measure the degree of similarity between two vectors of IBS proportions or IBD probabilities. The graphics we propose are of universal value and can be used in any relatedness study that concerns diploid individuals.

The remainder of this article is organized as follows. Section 2 gives an overview of the IBS allele sharing analysis and the graphical methods used to detect family relationships. Section 2.1 presents the basic principles of IBD estimation and the most common graphics used for relatedness estimation in the IBD context. The former sections also detail the graphical methods from the field of CoDA used in IBS‐IBD approaches: the ternary diagram and the scatterplot of log‐ratios. Section 2.2 presents a way to enhance IBS and IBD graphics with convex hulls that express the degree of uncertainty about a relationship. Section 2.3 presents a case study with individuals from the Maya population. Finally, Section 2.4 summarizes the principal conclusions of this article and the pros and cons of each graphical method are discussed.

## 2 IBS Studies

IBS studies disregard if the alleles for any diploid individual are derived from a common ancestor. IBS allele sharing concerns the number of matches between the alleles of the genotypes of two individuals. Two diploid individuals can share 0 (e.g., A1/A1 and A2/A2 or A1/A2 and A3/A3), 1 (e.g., A1/A1 and A1/A2 or A1/A2 and A1/A3) or 2 (e.g., A1/A1 and A1/A1) IBS alleles for a specific genetic marker, and we will refer to these as IBS counts. To detect family relationships in a given population of *n* individuals and *m* genetic markers, the number of matches between IBS alleles (the IBS counts) is considered for each pair of individuals across genetic markers. That is, we move from a data set of *n* individuals and *m* genetic markers to a data set of $\left(\begin{array}{c} n\\ 2 \end{array}\right)$ pairs of individuals with the information of the IBS counts for *m* genetic markers. There are different ways to deal with this type of data as described below. First, we focus on the plot of means and standard deviations of the IBS counts (Abecasis et al., 2001). Second, we detail the plot of the proportions of the IBS counts (Rosenberg, 2006). To conclude this section, graphics from CoDA (Aitchison, 1986; Pawlowsky‐Glahn & Buccianti, 2011) are presented.

To illustrate the different IBS graphics that are introduced in this Section, we use five pairs of individuals with the information of IBS counts and IBS proportions for 377 microsatellites (see Table 1). The individuals are from the Maya population which we will analyse in Section 2.3. We consider a parent‐offspring (PO) pair, a full‐sib (FS) pair, a half‐sib (HS), avuncular (AV) or grandparent‐grandchild (GG) pair, a pair of first cousins (FC) and a pair of unrelated individuals (UN). We discuss the different graphics in the sections below.

**Table 1:** Computations for five pairs of individuals from the Maya population. Mean and standard deviation of IBS counts, proportion of sharing 0, 1 and 2 IBS alleles (*p*_0_, *p*_1_, *p*_2_) and estimated Cotterman coefficients (${\hat{k}}_{0},{\hat{k}}_{1},{\hat{k}}_{2}$) are shown

Type of relative	IBS studies					IBD studies		
	Mean	Standard deviation	*p*_0_	*p*_1_	*p*_2_	${\hat{k}}_{0}$	${\hat{k}}_{1}$	${\hat{k}}_{2}$
PO	1.34	0.48	0.002	0.650	0.348	0.009	0.991	0.000
FS	1.32	0.60	0.073	0.532	0.395	0.214	0.617	0.169
HS, AV or GG	1.09	0.64	0.160	0.581	0.259	0.447	0.553	0.000
FC	1.00	0.67	0.225	0.546	0.229	0.657	0.343	0.000
UN	0.86	0.67	0.308	0.526	0.166	0.731	0.269	0.000

### 2.1 $\left(\overset{¯}{x},s\right)$‐plot

Let *x*_*ijk*_ be the number (0, 1 or 2) of shared IBS alleles between individual *i* and *j* for the genetic marker *k*. Abecasis et al. (2001) proposed to compute the mean (${\overset{¯}{x}}_{ij}$) and variance ($s_{ij}^{2}$) over *K* genetic markers. The plot ${\overset{¯}{x}}_{ij}$ versus *s*_*ij*_ reveals characteristic clusters that correspond to the different family relationships for a given population.

**Figure 1:**
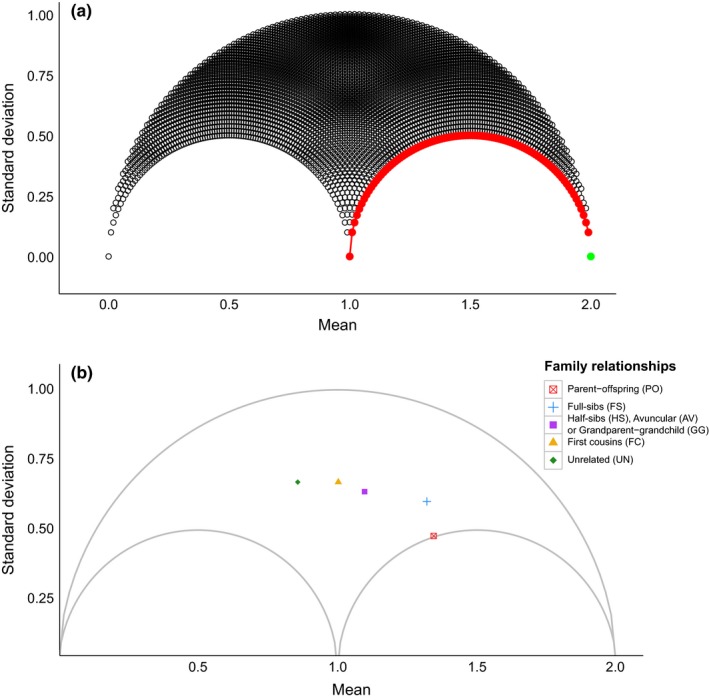
a. Plot of means and standard deviations of all possible combinations of IBS counts for a table of 100 genetic markers. The red curve shows the pairs of individuals that are parent‐offspring. The green point represents a monozygotic twin pair or a pair of duplicated individuals. b. Plot of means versus standard deviations of the IBS counts for five pairs from the Maya population

The statistics ${\overset{¯}{x}}_{ij}$ and $s_{ij}^{2}$ are constrained due to the limited number of outcomes (0, 1 or 2), and we proceed to derive their range of variation (Figure 1a). As an example, we consider a table with all possible outcomes of the allele sharing counts (0, 1 or 2) for a set of 100 markers. The rows of this table represent possible pairs of individuals. There are 3^100^ combinations (rows), if the order of the outcomes is considered relevant. However, in terms of means or standard deviations, the order of the IBS counts (0, 1 or 2) over the different markers is irrelevant but their multiplicity is important. For example, a pair of individuals sharing 1 IBS allele for the first marker and 0 for all other markers will have the same mean and variance as a pair of individuals sharing 1 IBS allele for the *k*‐th marker and 0 for all others. Mathematically, the combinations of the IBS counts for a pair of individuals form a multiset (Stanley, 1997, Section 1.2) of cardinality *m* (the number of markers) made of a basic set of cardinality *k* = 3 (the outcomes 0, 1 and 2). The possible number of ($\overset{¯}{x},s$) pairs in the plot can be no larger than the number of multisets of cardinality *k*, where the latter is given by the multiset coefficient

(1) $$\left(\left(\begin{array}{c} k\\ m \end{array}\right)\right)=\left(\begin{array}{c} k+m-1\\ k \end{array}\right),$$

Thus, for 100 genetic markers there will be at most $\left(\left(\begin{array}{c} 3\\ 100 \end{array}\right)\right)=\left(\begin{array}{c} 3+100-1\\ 100 \end{array}\right)=\left(\begin{array}{c} 102\\ 100 \end{array}\right)=5151$ different ($\overset{¯}{x},s$) pairs. Figure 1a shows the means and standard deviations of the 5151 combinations of IBS counts for 100 genetic markers. The figure has the shape of an umbrella and represent the domain of the ($\overset{¯}{x},s$)‐plot. For empirical data, it will be impossible to observe a $\left(\overset{¯}{x},s\right)$ point outside the umbrella region. It is clear that the mean of the IBS counts ranges from zero to two. The maximum variance equals one and is reached when the array of IBS counts has fifty 0 IBS alleles and fifty 2 IBS alleles, whereas the minimum variance equals zero and is reached when the array of IBS counts has either one hundred 0 IBS alleles, one hundred 1 IBS allele or one hundred 2 IBS alleles.

The red points on the right hand curve of the “umbrella” correspond presumably to parent–offspring relationships for having a mean larger than 1 and low variance. The first point of the curve with mean equal to 1 IBS allele and standard deviation equal to 0 IBS alleles corresponds to an array of one hundred ones. The second point of the curve corresponds to an array of 99 markers with 1 IBS alleles and one marker with 2 IBS alleles, and so on. In other words, this red curve represents the pairs of individuals who have a mean larger than or equal to 1 and the smallest standard deviation of all possible IBS counts. This can be related with the fact that the probability of sharing 1 IBD allele between a parent‐offspring equals 1, as we will see in the next Section (Table 2). For parent‐offspring pairs, we have that ${\overset{¯}{x}}_{ij}\geq 1$ because children inherit at least 1 IBS allele from their parents. And for monozygotic twins (MZ) or duplicated individuals, we have ${\overset{¯}{x}}_{ij}=2$ and *s*_*ij*_ = 0 (green point in Figure 1a).

**Table 2:** Cotterman coefficients for the different type of family relationship and degree of relatedness

Type of relative	Degree	*k*_0_	*k*_1_	*k*_2_
Monozygotic twins (MZ)	0	0	0	1
Parent‐offspring (PO)	1	0	1	0
Full‐siblings (FS)	1	1/4	1/2	1/4
Half‐siblings (HS)/avuncular (AV)/grandchild‐grandparent (GG)	2	1/2	1/2	0
First cousins (FC)	3	3/4	1/4	0
Unrelated (UN)	∞	1	0	0

Figure 1b shows the ($\overset{¯}{x},s$) plot for the five Maya pairs in Table 1. The larger the mean of the IBS counts for any pair of individuals, the more likely they are to be closely related. The PO pair (red point) is located on the right hand curve of the umbrella, the FS pair (blue point) with mean larger than 1 is separated from second‐ and third‐degree family relationships (violet and gold points respectively), whereas, the unrelated individuals have the smallest mean (green point).

### 2.2 (*p*_*i*_, *p*_*j*_)‐plots

Let *x*_*ij*_ be the vector of the IBS counts between individual *i* and *j* as large as the number of the genetic markers in the data set. Let *p*_0_, *p*_1_ and *p*_2_ be the proportions of 0, 1 and 2 IBS alleles, respectively, for each pair of individuals. Rosenberg (2006) proposed a graphical method for relatedness research by plotting the proportion of sharing 2 IBS alleles (*p*_2_) versus the proportion of sharing 0 IBS alleles (*p*_0_) for all pairs of individuals from a given population. Similarly, Sun (2012) uses IBS proportions for relatedness research by plotting *p*_1_ versus *p*_0_. In fact, any combination of the three proportions could be plotted for relatedness research. We refer to these graphics as (*p*_*i*_, *p*_*j*_)‐plots (for *i*, *j* = 0, 1, 2 and *i* < *j*) were *p*_*i*_ corresponds to the *X*‐axis of the plot and *p*_*j*_ to the *Y*‐axis.

**Figure 2:**
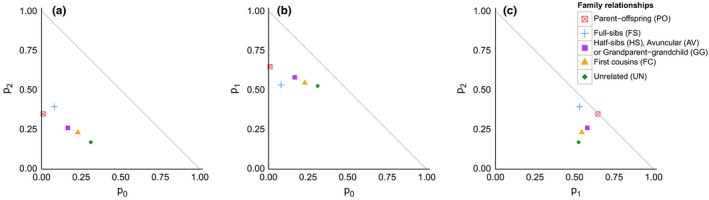
(*p*_*i*_, *p*_*j*_)‐plots for five individuals from the Maya population. a. Plot of the proportion of sharing 0 IBS alleles (*p*_0_) versus the proportion of sharing 2 IBS alleles (*p*_2_): (*p*_0_, *p*_2_)‐plot. b. Plot of the proportion of sharing 0 IBS alleles (*p*_0_) versus the proportion of sharing 1 IBS allele (*p*_1_): (*p*_0_, *p*_1_)‐plot. c. Plot of the proportion of sharing 1 IBS allele (*p*_1_) versus the proportion of sharing 2 IBS alleles (*p*_2_): (*p*_1_, *p*_2_)‐plot. [Colour figure can be viewed at wileyonlinelibrary.com]

Monozygotic twins (MZ) or duplicated individuals are easy to identify in the (*p*_*i*_, *p*_*j*_)‐plots because they have *p*_2_ close to 1. PO pairs have low values of *p*_0_ and are also easy to detect visually because they are on the *p*_1_ or *p*_2_‐axis. FS usually have large values of *p*_2_ and are separated from unrelated individuals. Second degree and third degree are more difficult to detect because positions in the plot depend on the allele frequencies of the population under study. Figures 2a, b and c show the (*p*_0_, *p*_2_)‐, (*p*_0_, *p*_1_)‐ and (*p*_1_, *p*_2_)‐plots for the five Maya pairs (Table 1). Notice that the distance between pairs of individuals is not the same in the three plots. For instance, the FS pair (blue point) is most close to the PO pair (red point) in the (*p*_0_, *p*_2_)‐plot, but closer to the HS pair (violet point) in the (*p*_0_, *p*_1_)‐plot. If the distances between pairs of individuals are different depending on the plotted proportions, then it is not appropriate to draw conclusions about the family relationship between individuals from the (*p*_*i*_, *p*_*j*_)‐plots.

### 2.3 Ternary diagrams

**Figure 3:**
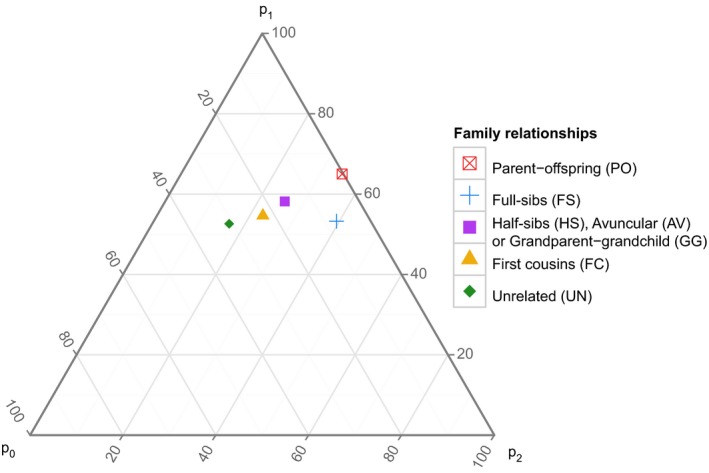
Ternary diagram of the IBS proportions for five pairs from the Maya population. [Colour figure can be viewed at wileyonlinelibrary.com]

Let ***p*** be the vector (*p*_0_, *p*_1_, *p*_2_) of proportions of the IBS counts. Because the three components of ***p*** sum to one (*p*_0_ + *p*_1_ + *p*_2_ = 1), we can plot the vector ***p*** in a ternary diagram. Mathematically, the set of the vectors of proportions ***p*** = (*p*_0_, *p*_1_, *p*_2_) forms the simplex, *S* ^3^. Figure 3 shows the ternary diagram for the vectors of proportions for the five Maya pairs (Table 1). The PO pair (red point) is located on the opposite side of the vertex *p*_0_; the FS pair (blue point) has the largest value for *p*_2_ and is the closest to the *p*_2_ vertex. The UN pair (green point), FC pair (gold point) and the HS, AV or GG pair (violet point) have lower values of *p*_2_. The UN pair has the lowest values for *p*_2_ and *p*_1_ and is closest to the *p*_0_ vertex. The main advantage of this graphical tool is that it represents the three proportions *p*_0_, *p*_1_ and *p*_2_ simultaneously in contrast to the (*p*_*i*_, *p*_*j*_)‐plots that represent only two of them.

### 2.4 ilr‐plots

Aitchison (1986) stated that it is not meaningful to interpret the distances between two vectors of proportions in the ternary diagram as if we were in an Euclidean space. Aitchison (1986) defines a new distance based on the log‐ratio of the components of the vectors of proportions. This distance, jointly with the perturbation and powering operators (analogous to translation and scalar multiplication in the real space, respectively), forms the structure of the simplex in a two‐dimensional metric space (Aitchison, Barceló‐Vidal, Martín‐Fernández, & Pawlowsky‐Glahn, 2000; Pawlowsky‐Glahn & Buccianti, 2011). Thereby, the vectors of proportions ***p*** = (*p*_0_, *p*_1_, *p*_2_) can be expressed in coordinates using any orthonormal basis defined in the simplex (Egozcue, Pawlowsky‐Glahn, Mateu‐Figueras, & Barceló‐Vidal, 2003). These coordinates are called isometric log‐ratio coordinates (ilr‐coordinates). The distance between two vectors of proportions is calculated as the Euclidean distance between their ilr‐coordinates. The ilr‐coordinates of a vector of proportions depend on the orthonormal basis used in the simplex. The most commonly used ilr‐coordinates ***z***_0_, ***z***_1_ and ***z***_2_ of a vector of proportions (*p*_0_, *p*_1_, *p*_2_) are given by

(2) $$z_{0}=\left\{\begin{array}{c} z_{01}=\frac{1}{\sqrt{2}}\ln\left(\frac{p_{2}}{p_{1}}\right)\\ z_{02}=\frac{1}{\sqrt{6}}\ln\left(\frac{p_{1}p_{2}}{p_{0}^{2}}\right) \end{array}\right.\;z_{1}=\left\{\begin{array}{c} z_{11}=\frac{1}{\sqrt{2}}\ln\left(\frac{p_{2}}{p_{0}}\right)\\ z_{12}=\frac{1}{\sqrt{6}}\ln\left(\frac{p_{0}p_{2}}{p_{1}^{2}}\right) \end{array}\right.\;z_{2}=\left\{\begin{array}{c} z_{21}=\frac{1}{\sqrt{2}}\ln\left(\frac{p_{1}}{p_{0}}\right)\\ z_{22}=\frac{1}{\sqrt{6}}\ln\left(\frac{p_{0}p_{1}}{p_{2}^{2}}\right) \end{array}\right.,$$

**Figure 4:**
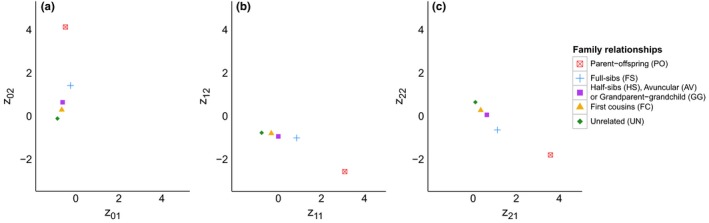
Ilr‐coordinates of the IBS proportions for five pairs of individuals from the Maya population. a. $z_{0}=\left(z_{01},z_{02}\right)$. b. $z_{1}=\left(z_{11},z_{12}\right)$. c. $z_{2}=\left(z_{21},z_{22}\right)$. [Colour figure can be viewed at wileyonlinelibrary.com]

Figures 4a, b and c plot the ilr‐coordinates for the five Maya pairs (Table 1). Notice that the distance between any pair of points is exactly the same in the three graphics, irrespective of the ilr‐coordinates (***z***_**0**_, ***z***_**1**_ and ***z***_**2**_) that are plotted. The PO pair (red point) in Figures 4a–c is an outlying pair. The FS pair (blue point) is also isolated from pairs of second and third degree of relationships. The degree of relationship decreases with the *z*_02_, *z*_11_ and *z*_21_ ilr‐coordinates (close relatives with a first‐degree relationship (PO, FS) have larger values for these coordinates than second‐degree relationships (HS, AV, GG)).

## 3 IBD Studies

Studies of relatedness based on IBD alleles are based on the probabilities that a pair of individuals shares 0, 1 or 2 IBD alleles. These probabilities are commonly referred to as Cotterman's coefficients (Cotterman, 1941) and denoted by the vector of proportions ***k*** = (*k*_0_, *k*_1_, *k*_2_). Table 2 shows the values of the Cotterman coefficients for some standard relationships. Cotterman's coefficients can be estimated by the maximum‐likelihood method (Milligan, 2003; Weir, Anderson, & Hepler, 2006). The maximum‐likelihood estimates reveal the most likely relationship for a pair given the observed genotype data. Let *R* represents a possible relationship between two individuals with genotypes *G*_1_ and *G*_2_, respectively. The likelihood of *R* is defined by the probability of observing *G*_1_ and *G*_2_ given relationship *R*. This probability depends on the allele frequencies of the population under study and is conditioned by the Cotterman coefficients. This likelihood is calculated across loci to obtain the most likely values (estimates) of the Cotterman coefficients. These estimates provide a first indication of the possible relationship between a pair of individuals. A hypothesis test is recommended to confirm or refute this relationship (García‐Magariños, Egeland, López‐de‐Ullibarri, Hjort, & Salas, 2015). More details are explained by Wagner, Creel, and Kalinowski (2006). Under the assumption of absence of inbreeding, the inequality $k_{1}^{2}\geq 4k_{0}k_{2}$ applies and constrains the Cotterman coefficients (Thompson, 1991).

Analogously to the vector of proportions ***p*** = (*p*_0_, *p*_1_, *p*_2_) of the IBS counts, Cotterman's coefficients also satisfy *k*_0_ + *k*_1_ + *k*_2_ = 1. We can use the same graphical techniques described for ***p*** = (*p*_0_, *p*_1_, *p*_2_) to identify relatedness from the estimated Cotterman coefficients $\hat{k}$. The Cotterman coefficients can be represented in a $\left({\hat{k}}_{i},{\hat{k}}_{j}\right)$‐plot, in a ternary diagram or in an ilr‐plot with the ilr‐coordinates ***z***_0_, ***z***_1_ and ***z***_2_, defined in the Equation (2), substituting *p*_*i*_ for ${\hat{k}}_{i}$. With the aim of describing each graphical method used in IBD studies, we compute maximum‐likelihood estimates of the Cotterman coefficients for the five Maya pairs (Table 1).

### 3.1 $\left({\hat{k}}_{i},{\hat{k}}_{j}\right)$‐plots

**Figure 5:**
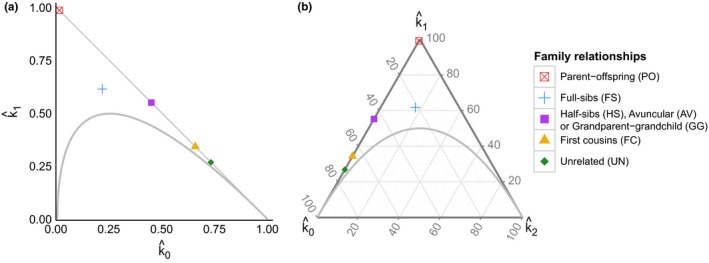
(${\hat{k}}_{0}$, ${\hat{k}}_{1}$)‐plot (a) and ternary diagram (b) for five pairs of individuals from the Maya population. [Colour figure can be viewed at wileyonlinelibrary.com]

In the literature, the estimated Cotterman coefficients are plotted in different ways to identify relatedness. Nembot‐Simo, Graham, and McNeney (2013) use the $\left({\hat{k}}_{0},{\hat{k}}_{1}\right)$‐plot. Similarly, Moltke and Albrechtsen (2014) use the $\left({\hat{k}}_{1},{\hat{k}}_{2}\right)$‐plot. The remaining possibility, the $\left({\hat{k}}_{0},{\hat{k}}_{2}\right)$‐plot, could be also considered. Figure 5a shows the plot for the five Maya pairs (Table 1). The grey curve in the $\left({\hat{k}}_{0},{\hat{k}}_{1}\right)$‐plot corresponds to the equation $k_{1}^{2}=4k_{0}k_{2}$. This curve jointly with the hypotenuse and the vertical axis delimits the feasible region $k_{1}^{2}\geq 4k_{0}k_{2}$. PO pairs are points located on the *k*_1_‐axis with values close to 1, FS pairs are located close to the centre of the grey curve according to the theoretical IBD probabilities (Table 2) and second and third degree pairs are located around the centre of the hypotenuse. UN pairs theoretically have *k*_0_ = 1 and are located between the hypotenuse and the grey curve, near to the vertex ${\hat{k}}_{0}=1$. Finally, the origin of the $\left({\hat{k}}_{0},{\hat{k}}_{1}\right)$‐plot is the position for any MZ pair. As previously shown for IBS studies with the (*p*_*i*_, *p*_*j*_)‐plots, only two of the three Cotterman coefficients are plotted and the relative positions and distances between points vary depending on the (${\hat{k}}_{i},{\hat{k}}_{j}$)‐plot used. For this reason, we propose graphics from CoDA.

### 3.2 Ternary diagrams

The theoretical IBD probabilities for the standard family relationships can be represented in a ternary diagram (Thompson, 2000). These probabilities form reference points against which the empirical estimates can be compared. Figure 5b shows the ternary diagram for the estimated Cotterman coefficients for the five Maya pairs (Table 1). Most pairs in Table 1 are close to their theoretical IBD probabilities given in Table 2. However, values of *k*_1_ are larger than expected for the FS, HS, AV and notably, the UN pair (see the Discussion section). The domain has the shape of an arrowhead inside the ternary diagram. The curve delimiting the arrowhead from below corresponds to the inequality $k_{1}^{2}\geq 4k_{0}k_{2}$.

### 3.3 ilr‐plots

**Figure 6:**
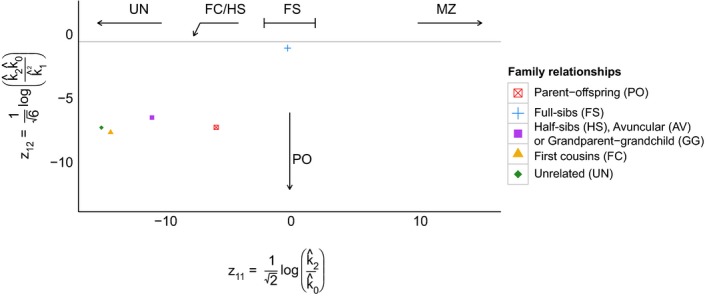
Ilr‐coordinates $z_{1}=\left(z_{11},z_{12}\right)$ of the estimated Cotterman coefficients (${\hat{k}}_{0}$,${\hat{k}}_{1}$,${\hat{k}}_{2}$) for five pairs of individuals from the Maya population. [Colour figure can be viewed at wileyonlinelibrary.com]

It has been shown that the maximum‐likelihood estimates of the Cotterman coefficients in the simplex are the same as the estimates obtained by maximizing the likelihood in ilr‐coordinates (Graffelman & Galván‐Femenía, 2016). With the aim of establishing reference zones for the standard family relationships in the ilr space, we compute the maximum‐likelihood estimates of the Cotterman coefficients from the ilr‐coordinates defined by the Equation (2) and we plotted the $z_{1}=\left(z_{11},z_{12}\right)$ ilr‐coordinates as is shown in Figure 6. All the family relationships have values lower than $-\sqrt{\left(2/3\right)}\ln\left(2\right)$ for $z_{12}$ which corresponds to the grey line in the graph. This line corresponds to the curve shown in the former graphs (Figure 5a and b). Due to the fact that some Cotterman coefficients equals 0, some of the (or both) ilr‐coordinates tend to +/−  infinity. Thus, given that it is impossible to represent the point, we are limited to indicate the direction of the infinity in the ilr‐plot for each type of family relationship. Regarding Figure 6, PO pairs have a large variability of values, either positive or negative for *z*_11_; FS have values close to 0 for *z*_11_ and $-\sqrt{\left(2/3\right)}\ln\left(2\right)$ for *z*_12_. HS, AV, GG and FC are located between PO, FS and UN. UN pairs have negative values of *z*_11_ which correspond to the green point of the left hand. If present, MZ pairs are points with positive values of *z*_11_ located on the right hand side of the plot.

## 4 Uncertainty in IBS/IBD graphics

With the previously described graphics, one can try to infer the relationship of a pair for which the relationship is not documented, or try to confirm the documented relationships. Such graphical inference is hampered by the fact that the statistics represented in the graphs (means and standard deviations of the IBS counts, *p*_0_, *p*_1_, *p*_2_, *k*_0_, *k*_1_, *k*_2_) are subject to uncertainty. For a given sample, relationships are not represented by points, but by zones. Some insight into this uncertainty and the corresponding zones can be obtained by simulation. Ideally, this would require a large sample for which a subset of unrelated individuals can be identified. From these individuals, by sampling alleles across markers according to Mendelian laws, the reproductive process can be simulated allowing us to generate artificial children, leading to artificial PO pairs, FS pairs and artificial pairs of any other desired relationship. For example to simulate a PO pair we sample two UN individuals at random without replacement from the database. From each UN individual, we sample one allele at random from each marker and join the alleles to form a child. The process of sampling UN pairs and child generation is repeated many times, generating many artificial PO pairs. We can calculate the IBS/IBD statistics of the artificial pairs, and add these to the graphics of the previous sections by representing them individually or with a convex hull. A convex hull for a given set of points *X* is the unique convex polygon whose vertices are points from *X* and that contains all points of *X* (de Berg, van Kreveld, Overmars, & Schwarzkopf, 2000). By generating a large number of artificial pairs and representing these in the IBS/IBD graphics of interest, the zones corresponding to the different relationships can be approximated. Such simulations are conditional on the observed allele frequencies and can quantify the uncertainty in a graphical assessment of the relationship to some extent. We illustrate this with examples in the next section where all graphics are enhanced with hulls based on 80 PO, 48 FS, 120 second degree, 36 FC and 1256 UN artificially generated pairs.

## 5 Case study

We applied all the graphical methods detailed in the previous sections using empirical data extracted from a world‐wide data set from the Noah A. Rosenberg Research lab at Stanford University (Rosenberg et al., 2002). This world‐wide database is derived from the Human Genome Diversity Cell Line Panel (HGDP, Cavalli‐Sforza, 2005). The genetic information is given by 377 microsatellites genotyped for 52 human populations around the world. We used all 25 available individuals of the Maya sample to illustrate all graphical methods for relatedness research. All the family relationships present in this sample were reported by Rosenberg (2006). All the Figures presented throughout this article are made with the R software (R Core Team, 2015) using the R packages **ggplot2** (Wickham, 2009) and **ggtern** (Hamilton, 2015).

### 5.1 IBS graphics

**Figure 7:**
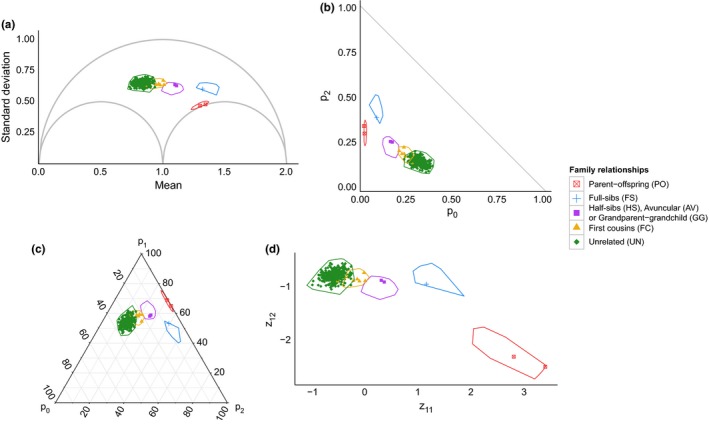
Identical by state (IBS) alleles for all the pairs of individuals from the Maya population. a. Plot of means versus standard deviations. b. (*p*_2_, *p*_0_)‐plot. c. Ternary diagram. d. Ilr‐coordinates: $z_{1}=\left(z_{11},z_{12}\right)$. The convex hulls are obtained by simulating artificial children from a subset of unrelated individuals from the Maya population and each hull is based on 80 PO, 48 FS, 120 second degree, 36 FC and 1256 UN artificial pairs

Figure 7 shows all IBS graphics for all pairs of the Maya population. In the $\left(\overset{¯}{x},s\right)$‐plot (Figure 7a), the points with the smallest standard deviation close to the grey curve are two PO pairs. The relationships of first and second degree are the points with a mean above 1. Note that some pairs of FC are mixed with UN pairs. Figure 7b (the (*p*_0_, *p*_2_)‐plot) clearly separates the family relationships of first and second degree from the UN pairs. In the ternary diagram (Figure 7c), PO pairs are points on the opposite side of the vertex *p*_0_, meaning that the *p*_0_ is close to 0. The FS pair is the point closest to the vertex *p*_2_, which has the largest *p*_2_; the violet points represent the family relationships of second degree are separated from the green points representing UN pairs. In Figure 7d, the first ilr‐coordinate (*z*_11_) clearly discriminates first‐degree relatives from UN pairs. Pairs with larger values for *z*_11_ are more likely to correspond to related individuals. PO pairs are extreme outliers because they have *p*_0_ values close to 0 which increase the first coordinate of the corresponding log‐ratio. The scatterplot of the log‐ratios is seen to produce a larger degree of separation between FS and PO pairs, and between first‐degree relationship pairs and all other pairs. The convex hulls for the simulated related pairs in Figure 7 are seen to enclose the sample estimates of the PO, FS, HS and FC pairs and so confirm the assigned relationships.

### 5.2 IBD graphics

**Figure 8:**
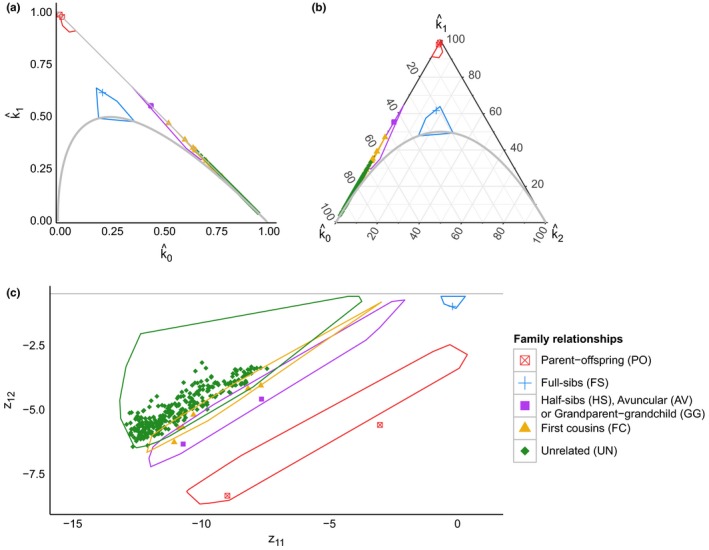
Identical by descent (IBD) alleles for all the pairs of individuals from the Maya population. a. $\left({\hat{k}}_{0},{\hat{k}}_{1}\right)$‐plot. b. Ternary diagram. c. Ilr‐coordinates: $z_{1}=\left(z_{11},z_{12}\right)$

We estimated IBD probabilities for all pairs of the Maya population. All IBD graphics are shown in Figure 8. The $\left({\hat{k}}_{0},{\hat{k}}_{1}\right)$‐plot (Figure 8a) separates the first, second and some pairs of third degree of relatedness. In the ternary diagram of $\hat{k}$ (Figure 8b), it is easy to identify PO pairs at the vertex of ${\hat{k}}_{1}$, a FS pair close to the barycenter of the triangle and other family relationships of second degree on the opposite side of the ${\hat{k}}_{2}$ vertex. UN pairs are on the *k*_0_ – *k*_1_ edge and tend towards the *k*_0_ vertex. Third‐degree pairs are mixed with unrelated individuals. In the ilr‐plot (Figure 8c), the pairs with a close family relationship tend to have larger values of *z*_11_. The family relationships of the first degree (FS and PO) are located according to the directions indicated in Figure 6. The ilr‐plot clearly separates out these FS and PO relationships from all other pairs. Notice that Figure 8a and b show only one pair with a second degree relationship (the violet point), whereas in Figure 8c, there are two visible violet pairs. The IBD graphics were also amplified with convex hulls of artificially generated related pairs to show the approximate expected positions for the different relationships. These hulls mainly confirm the assigned relationships. In ilr‐coordinates, PO hulls do not capture all observed PO pairs (see Discussion).

## 6 Discussion

The main aim of this article was to review all graphical methods used in relatedness research. We have distinguished graphics based on IBS and IBD allele sharing. Plotting means versus standard deviations of the IBS counts allows us to detect monozygotic twins (MZ), parent‐offspring (PO) and full‐sibs (FS) pairs. However, higher degree relationships are more difficult to detect visually. The distances between unrelated and related pairs depend on the allele frequency distribution of the markers under study. The larger the heterozygosity in a population, the larger the distances between related and unrelated individuals are. A disadvantage of this mean‐variance plot is that there are no fixed reference points for the standard relationships. Such reference points could eventually be found by calculating expectations of the mean and the variance of the IBS counts. These do depend on the allele frequency distribution and will therefore depend on the population that has been sampled, and on the distribution of the allele frequencies in that population. The (*p*_*i*_, *p*_*j*_)‐plots allow easy detection of MZ pairs (or duplicated individuals) because they have *p*_2_ values close to 1, and PO pairs have low values of *p*_0_ and are also easy to detect. FS pairs are located between PO pairs and the pairs with large values of *p*_0_. However, it remains hard to detect relationships of the second and third degree. The (*p*_*i*_, *p*_*j*_)‐plots neither have a fixed reference position for the standard relationships. Moreover, as has been noted in Section 2, the Euclidean distance between two pairs in a (*p*_*i*_, *p*_*j*_)‐plot is not invariant with respect to the chosen index (0, 1 or 2), for example, is not the same in a (*p*_0_, *p*_1_) and a (*p*_0_, *p*_2_)‐plot. $\left({\hat{k}}_{i},{\hat{k}}_{j}\right)$‐plots have, in comparison with (*p*_*i*_, *p*_*j*_)‐plots, the advantage that fixed reference positions for the standard relationships exist, as given in Table 2. This is of great practical value when inferring relationships. Moreover, IBD plots are more reliable for classifying relationships because they show a larger degree of separation between the different relationships than their IBS counterparts. This is clearly visible when one compares Figures 2 with 5a, 3 with 5b, 7b with 8a and 7c with 8b. However, the IBD‐based $\left({\hat{k}}_{i},{\hat{k}}_{j}\right)$‐plots suffer from the same problem as their IBS counterparts: the Euclidean distances between pairs (and reference points) depend on the index (0, 1 or 2) that is used.

We comment on some peculiarities of the HGDP‐CEPH database analysed in the article. We found the high estimate of *k*_1_ (0.27) in Table 1 for the reported UN pair to be not too unusual for Maya UN pairs, being the median of *k*_1_ 0.17 for UN pairs of this population. The relatively high *k*_1_ estimates are probably to some extent due to inbreeding, as the South American populations had the largest medians of *k*_1_ for UN pairs. However, for many other less inbred populations *k*_1_ estimates of UN pairs had a large median too, in the range 0.1–0.2. We suggest the database could be affected by a certain degree of sample contamination, as this will increase the number of heterozygote calls, leading to overestimated IBD (Andoh, Sato, Sakamoto, Yoshida, & Ohtaki, 2010).

We continue with some remarks on the graphics from CoDA proposed in this article. We advocate the ternary diagram as an alternative for the (*p*_*i*_, *p*_*j*_)‐plots because it clearly shows all three proportions simultaneously. MZ twins are close to the vertex *p*_2_; PO pairs are easy to identify on the opposite side of the vertex *p*_0_. FS pairs usually have large values of *p*_2_ and are separated from unrelated pairs which have lower values of *p*_2_. We also advocate the ternary diagram for IBD studies for the same reasons: all three estimated IBD probabilities are represented in one single graph with all three ${\hat{k}}_{i}$ axes. The theoretical IBD probabilities (Table 2) are easily added for use as reference points. The ternary diagram resolves the indeterminacy of the Euclidean distances between pairs due to the choice of axes observed above in (*p*_*i*_, *p*_*j*_) and (*k*_*i*_, *k*_*j*_) scatterplots. However, the interpretation of Euclidean distances in the ternary diagram remains a tricky issue, because the simplex is a constrained space. We note that the Euclidean distance is regarded inadequate for the comparison of compositions, and for this reason, we have considered isometric log‐ratio coordinates of IBS and IBD probabilities. The Euclidean distances between the pairs in ilr‐coordinates correspond to Aitchison distances between (*p*_0_, *p*_1_, *p*_2_) (or (*k*_0_, *k*_1_, *k*_2_)) compositions. The Aitchison distance is considered to be an adequate metric for representing compositions (Pawlowsky‐Glahn, Egozcue, & Tolosana‐Delgado, 2015, Chapter 3). Plotting the ilr‐coordinates of the IBS proportions is useful for detecting related individuals because usually unrelated individuals are concentrated in a cloud of points and most outlying individuals correspond to related pairs. Plotting the ilr‐coordinates of the estimated Cotterman coefficients gives reference zones over the ilr space for the different relationships (Figure 6). Standard family relationships can be inferred depending on the values of $z_{11}$ and $z_{12}$. UN pairs are mainly represented in the scatterplot of the isometric log‐ratios of IBD probabilities by a central cloud of points around (−10, −5) (Figure 8c) but also by points close to the upper limit of the second ilr‐coordinate ($-\sqrt{\left(2/3\right)}\ln\left(2\right)$). A small change in the tolerance or the initial point of the maximization algorithm can greatly influence the final position of an UN pair. Both IBS‐ and IBD‐based log‐ratio plots show a strong discrimination of PO and FS pairs which typically appear as outliers in these plots. We also note that all inference on relationships in all presented graphical methods relies on the judgement of the analyst, who interprets distances between points in a graph. Depending on the sample size of the study, the number of markers used for the genotyping and the distributions of their allele frequencies, those distances will be subject to some degree of uncertainty which complicates graphical inference on relationships. By simulating artificial related pairs using the genotypes of unrelated pairs of the database, convex hulls for the expectation of the standard relationships can be obtained, which are conditional on the observed sample allele frequencies. These convex hulls assess the degree of uncertainty that can be expected for the different related pairs and are helpful for confirming putative relationships. In the present work, the convex hulls are limited by the fact that they assumed independent markers. This may explain why some related pairs are outlying with respect to their corresponding convex hulls. The accuracy of the convex hulls depends on the sample size, and in particular on the number of UN individuals in the sample from which it is generated. More accurate convex hulls may be obtained if linkage disequilibrium is taken into account and artificial pairs are generated by sampling from haplotypes instead of by sampling individual markers independently. Convex hulls of PO pairs in ilr‐coordinates often do not capture all observed PO pairs (Figure 8). We suggest this might be due to a small sample size combined with numerical instability. The position of a PO pair in ilr‐coordinates has a high variability and depends on the tolerance and initial point used in the maximization of the likelihood (Graffelman & Galván‐Femenía, 2016). If the sample size is small, or the number of simulated pairs is small, the PO hull many not cover the full area compatible with PO pairs. It is worth remarking that PO and FS convex hulls do not intersect each other and do not overlap with the rest of the hulls, having a valuable discrimination power (Figures 7 and 8). We think the current simulated convex hulls are helpful to assess uncertainty but of limited value and see a clear need for methods of formal statistical inference on relationships by means of hypothesis testing and confidence regions (García‐Magariños et al., 2015).

## 7 Software

R functions for making the graphics in this manuscript are available from the Dryad Digital Repository: https://doi.org/10.5061/dryad.2532d.

## Author Contributions

All authors contributed to the writing of this article. I.G.F. analysed data.

## Data accessibility

The data analysed in this article are freely available on the web of the Rosenberg lab at Stanford University (https://rosenberglab.stanford.edu/diversity.html\#2002).
