# Supplementary material for: Graphics for relatedness research
Source: Mol Ecol Resour. 2017 May 12;17(6):1271–82. doi: 10.1111/1755-0998.12674 (PMC5624821; doi:10.1111/1755-0998.12674)
Supplement: Supplementary file 1 [file MEN-17-1271-s001.docx]

Supporting information

Figure S1. IBS graphics for the Maya, Colombian and Yoruba populations. a. Means versus standard deviations of the IBS counts. The vertical green line separates the unrelated individuals from the related individuals. b. $(p_0,p_2)$-plot.

Figure S2. IBS graphics for the Maya, Colombian and Yoruba populations. c. Ternary diagram of $(p_0,p_1,p_2)$. d. Ilr-coordinates: $ {\bi z_1} = (z_1_1,z_1_2)$.

Figure S3. IBD graphics for the Maya, Colombian and Yoruba populations. a. $(\hat{k}_0,\hat{k}_1)$-plot. b. Ternary diagram of the estimated Cotterman coefficients.

Figure S4. IBD graphics for the Maya, Colombian and Yoruba populations. c. Ilr-coordinates $ {\bi z_1} = (z_1_1,z_1_2)$.
